# Supplementary material for: Latino/a experiences of homelessness in California: Qualitative findings from the California Statewide Study of People Experiencing Homelessness (CASPEH)
Source: PLoS One. 2026 Mar 5;21(3):e0344036. doi: 10.1371/journal.pone.0344036 (PMC12962465; doi:10.1371/journal.pone.0344036)
Supplement: S1 Data — (DOC) [file pone.0344036.s001.doc]

|  |  |
| --- | --- |

*For questions related to data access, please contact* [*research@ucsf.edu*](mailto:research@ucsf.edu)*.*
