# Supplementary material for: Latino/a experiences of homelessness in California: Qualitative findings from the California Statewide Study of People Experiencing Homelessness (CASPEH)
Source: PLoS One. 2026 Mar 5;21(3):e0344036. doi: 10.1371/journal.pone.0344036 (PMC12962465; doi:10.1371/journal.pone.0344036)
Supplement: Attachment — Submitted filename: plos_one_rvw_D_25_27066.docx [file pone.0344036.s003.docx]

Latino/a experiences of homelessness in California: Qualitative findings from the California Statewide Study of People Experiencing Homelessness (CASPEH)

This is a well done and informative study to identify and describe drivers of Latino/a homeless in California (CA). Qualitative interviews of 84 participants from the larger CASPEH study provide the data. The introduction makes a compelling case for Latino/a homelessness as a growing public health problem. Authors do a good job laying out challenges to sampling CA Latino/as in page 4 and justification for venue based sampling supplemented by respondent driven sampling.

More detail on how “purposive sampling” which should be defined (lines 127-128) is used to choose 365 participants for qualitative interviews from the 3,200 is needed. Authors should address “purposive sampling” not leading to bias. The sample for this manuscript (Lines 141-143) comes from Latino/as from one of 7 interview based sub-studies. Some basic demographic information about similarities and differences of Latinos in these different sub-studies would be helpful. For example, what percentage of interviews were conducted in Spanish in each of the seven sub-studies as a marker of acculturation, age, gender, birth outside US?

The term “housing returns” in abstract line 45 and line 125 should be defined. Perhaps this means an inability to return to temporary housing?

The interview quotes in the Results section are illustrative and support the themes under which they are grouped.

Lines 459-461- I would add word “negatively”: “Our study took place prior to the Trump administration’s second term, during which several policies have shifted in ways that negatively directly impact this population.”

This is a timely qualitative study with valuable difficult to obtain interview data on drivers of Latino/a homelessness in the US’ most populous state. It is an important contribution to the public health and social science literature.
